# Construction and Characterization of a Vesicular Stomatitis Virus Chimera Expressing Schmallenberg Virus Glycoproteins

**DOI:** 10.3390/vetsci12090809

**Published:** 2025-08-25

**Authors:** Huijuan Guo, Zhigang Jiang, Jing Wang, Fang Wang, Qi Jia, Zhigao Bu, Xin Yin, Zhiyuan Wen

**Affiliations:** State Key Laboratory for Animal Disease Control and Prevention, Harbin Veterinary Research Institute, Chinese Academy of Agricultural Sciences, Harbin 150069, China; 18738046032@163.com (H.G.); jiangzhigang@caas.cn (Z.J.); wangjing_19940223@163.com (J.W.); wangfang06@caas.cn (F.W.); jiaqivet@163.com (Q.J.)

**Keywords:** Schmallenberg virus, envelope glycoproteins, vesicular stomatitis virus, virus neutralization test

## Abstract

Schmallenberg virus (SBV) is a negative-sense RNA virus transmitted by insect vectors, causing arthrogryposis-hydranencephaly syndrome in newborn ruminants. Since its discovery in Germany and the Netherlands in 2011, SBV has rapidly spread across multiple European countries, resulting in significant economic losses in the livestock industry. With the increasing global animal trade and the expanded range of insect transmission, the risk of SBV introduction into non-endemic regions is also rising. As the gold standard for serological testing, the virus neutralization test (VNT) is crucial for tracking the spread of SBV and evaluating the efficacy of vaccines. However, in non-endemic regions, the lack of local viral strains and the biosafety risks associated with introducing foreign strains pose challenges to the implementation of VNT. In this study, we employed reverse genetics techniques using vesicular stomatitis virus (VSV) to substitute the VSV G protein with the envelope glycoproteins of SBV, thereby successfully generating and rescuing the recombinant virus rVSVΔG-eGFP-SBVGPC. The recombinant virus was then thoroughly characterized in terms of SBV Gc protein expression, viral morphology, and growth kinetics. Importantly, rVSVΔG-eGFP-SBVGPC exhibited SBV-specific cell tropism and was capable of reacting with SBV-positive serum, enabling the measurement of neutralizing antibody titers. The results suggest that this recombinant virus can serve as a feasible alternative for SBV neutralization tests, with promising potential for application in serological screening and vaccine evaluation.

## Simple Summary

Schmallenberg virus (SBV) is an insect-borne virus that can cause severe deformities in newborn ruminants, leading to significant economic losses. Since its first detection in Germany and the Netherlands in 2011, SBV has rapidly spread across Europe and poses a risk of introduction into non-endemic regions. Detection of SBV infection often relies on the virus neutralization test (VNT), which requires live virus and faces challenges of availability and biosafety in non-affected areas. In this study, we used reverse genetics to construct a safe recombinant vesicular stomatitis virus (VSV) displaying SBV envelope proteins. This engineered virus can mimic the interaction between SBV and antibodies, enabling the detection of neutralizing antibodies without the use of the authentic virus, thus providing a safer and more practical alternative for SBV surveillance and vaccine evaluation.

## 1. Introduction

Schmallenberg virus (SBV) is an emerging orthobunyavirus affecting ruminants, first detected in dairy cattle in Schmallenberg, North Rhine-Westphalia, Germany, in 2011 [1]. Since then, the virus has rapidly spread across several European countries, presenting a significant threat to the livestock industry [2]. SBV is primarily transmitted by insect vectors, particularly midges and mosquitoes, and is closely linked to outbreaks of abortion, stillbirths, and congenital malformations in affected animals [3,4,5]. The transmission dynamics of SBV are complex, influenced by environmental factors, host immune responses, and vector activity. The virus has a wide host range, infecting cattle, sheep, goats, and wild ruminants such as deer, with sporadic detections in other species, including dogs. SBV has expanded its geographic reach beyond Europe, spreading to parts of Africa and western Asia [6,7,8,9,10,11], resulting in substantial economic losses in the livestock sector.

The genome of SBV comprises three negative-sense single-stranded RNA segments: small (S), medium (M), and large (L) [12]. The L segment encodes RNA-dependent RNA polymerase, which is essential for viral replication. The S segment encodes the nucleocapsid (N) protein and non-structural (NS) protein, while the M segment encodes a glycoprotein precursor protein (GPC), which is cleaved during co-translational processing to generate two essential envelope glycoproteins (Gn and Gc) and non-structural protein NSm [13,14,15]. The Gn protein (32–35 kDa) facilitates viral attachment by binding to host cell receptors, whereas the Gc protein (100–110 kDa) mediates membrane fusion, allowing the viral genome entry into the cytoplasm [16,17,18]. Notably, the Gc protein plays a crucial role not only in viral infection but also in inducing neutralizing antibody responses, making it a major target for vaccine development and serological detection [18,19,20]. Ongoing studies are driving advances in SBV vaccine design and the optimization of surveillance methods. These efforts enhance SBV monitoring, mitigate economic losses in the livestock industry, and support public health safety by preventing potential zoonotic transmission.

As an emerging pathogen, the initial outbreaks of SBV were concentrated in Europe. However, climate change may influence its epidemiology, facilitating its spread to new geographical regions [21,22]. Recent studies have, for the first time, detected SBV antibodies in ruminants in southern China, indicating the necessity of enhanced surveillance [23]. Establishing effective monitoring systems in newly affected regions, including China, is of great importance for the early detection and timely control of outbreaks. The virus neutralization test (VNT) is considered the “gold standard” for detecting SBV antibodies, as it precisely measures neutralizing antibody titers, directly reflecting the host’s immune response. Although time-consuming, VNT offers superior sensitivity and specificity compared to other serological tests, particularly in distinguishing between acute infection and past exposure, making it a reliable diagnostic tool. However, in countries where SBV is not endemic, VNT implementation is limited due to the need for circulating viral strains, which poses challenges for early detection, as well as vaccine development and evaluation.

Vesicular stomatitis virus (VSV) is a bullet-shaped, single-stranded RNA virus from the Rhabdoviridae family, encoding five structural proteins: nucleoprotein (N), phosphoprotein (P), matrix protein (M), glycoprotein (G), and RNA-dependent RNA polymerase (L) [24]. The G protein plays a critical role in receptor binding and membrane fusion [25]. Due to its simple and manipulable genome, VSV can be genetically modified using reverse genetics to generate recombinant VSV (rVSV), replacing its G protein with that of other enveloped viruses. This enables the expression of foreign viral proteins on its surface, mimicking the entry process of target viruses and inducing specific immune responses [26,27,28]. As a result, VSV serves as an ideal tool for studying heterologous viral glycoproteins.

In this study, we employed a reverse genetic system to generate a VSV chimaera, rVSVΔG-eGFP-SBVGPC, in which the native VSV G gene was replaced with an enhanced green fluorescent protein (eGFP) reporter gene and the M gene segment of SBV. The rVSVΔG-eGFP-SBVGPC replicates efficiently in Vero cells and successfully incorporates SBV envelope glycoproteins into its viral particles. This chimeric virus successfully recapitulates the key infection characteristics of SBV and can serve as a valuable tool for antibody detection and vaccine development. Our findings contribute to a deeper understanding of SBV, providing a scientific foundation for improved surveillance, early detection, and control strategies in China and other non-endemic regions.

## 2. Materials and Methods

### 2.1. Cells, Virus and Sera

African green monkey kidney cells (Vero, ATCC, CRL-1586), human embryonic kidney 293T cells (HEK293T, ATCC, CRL-3216), baby hamster kidney stably transfected cells (BSR, CVCL_RW96), porcine kidney 15 cells (PK-15, ATCC, CCL-33), Madin–Darby canine kidney cells (MDCK, ATCC, CCL-34), Madin–Darby bovine kidney cells (MDBK, ATCC, CCL-22), Madin–Darby ovine kidney cells (MDOK, ATCC, CRL-1633), and bovine turbinate cells (BT, ATCC, CRL-1390) were maintained in Dulbecco’s modified Eagle’s medium (DMEM) (Gibco, Waltham, MA, USA) supplemented with 10% fetal bovine serum (FBS) (ExCell, Shanghai, China), 1% L-glutamine, and 1% penicillin-streptomycin. All cells were incubated at 37 °C with 5% CO_2_ to ensure optimal growth conditions. The recombinant Indiana strain vesicular stomatitis virus expressing enhanced green fluorescent protein (rVSV-eGFP) was constructed and preserved in our laboratory. Additionally, a mouse-derived anti-SBV Gc serum was prepared for further analyses. SBV-positive freeze-dried bovine serum was purchased from Innovative Diagnostics (catalog number MRI-SBV). Fetal bovine serum (FBS) was used as a negative control in the neutralization assays to establish baseline values for comparison.

### 2.2. Plasmids

The VSV genome plasmid and helper plasmids, including pCI-VSV G, pCI-VSV L, pCI-VSV N, pCI-VSV P, and pCI-VSVΔG-eGFP, were constructed and are preserved in our laboratory. The SBV M gene segment (GenBank: KC355458.1) was synthesized and codon-optimized for mammalian cells, then subcloned into the NheI restriction site of the pCI vector, resulting in the construction of the pCI-VSVΔG-eGFP-SBVGPC plasmid. All plasmid constructs were confirmed through Sanger sequencing to ensure accuracy and integrity.

### 2.3. Rescue of the rVSVΔG-eGFP-SBVGPC Virus

BSR cells were cultured in DMEM supplemented with 10% fetal bovine serum (FBS). When the cell density reached approximately 80%, plasmid transfection was performed using a lipid-based transfection method. The plasmids were mixed in the following ratio: pCI-VSVΔG-eGFP-SBVGPC (2 μg), pCI-VSV-N (2 μg), pCI-VSV-P (2 μg), pCI-VSV-L (1 μg), and pCI-VSV-G (1 μg), with a total plasmid amount of 8 μg per well. The plasmid mixture was incubated with an appropriate amount of X-tremeGENE HP DNA Transfection Reagent for 20 min before being added to the cell culture medium for transfection. Six hours post-transfection, the medium was replaced with fresh DMEM containing 10% FBS, and incubation continued. Green fluorescence typically appeared 72 h post-transfection. When over 90% of the cells exhibited green fluorescence, the culture supernatant was collected and passed onto Vero cells for amplification. Upon observation of significant cytopathic effects (CPE), with more than 90% of the cells infected, the recombinant virus rVSVΔG-eGFP-SBVGPC was harvested and stored at −80 °C for subsequent use.

### 2.4. Virus Purification

BSR cells were seeded at a density of 30% in a T225 cell culture flask and incubated at 37 °C with 5% CO_2_ for 10–12 h until the cell confluence reached 90%. After discarding the DMEM, the cells were washed three times with PBS, then infected with rVSV-eGFP and rVSVΔG-eGFP-SBVGPC at an MOI of 0.1 for 1 h. Afterward, cells were cultured in DMEM supplemented with 2% FBS. After 48 h, virus-infected cells and culture supernatant were collected, and cell debris was removed by centrifugation at 2000 rpm for 20 min at 4 °C. The virus was inactivated by incubation with β-propiolactone at a ratio of 1:3000 (*v*/*v*) at 4 °C overnight, followed by treatment at 37 °C for 2 h. The inactivated virus was purified and concentrated using a discontinuous sucrose density gradient (20% to 60%) centrifugation at 80,000× *g* for 2 h at 4 °C. The virus band was collected, dissolved in PBS, and stored at −80 °C for future use.

### 2.5. Western Blotting

BSR cells were cultured in DMEM containing 10% FBS until the cell density reaches 80–90%. After removing the medium, the cells were infected with either rVSVΔG-eGFP-SBVGPC or rVSV-eGFP virus (MOI 0.1) and incubated at 37 °C for 1 h. Following infection, the cells were cultured for an additional 36 h. The infected cells were then lysed with NP-40 lysis buffer at 4 °C for 1 h. SDS-PAGE analysis was performed to separate proteins, followed by transfer to a membrane. The membrane was blocked with 5% skim milk and incubated with mouse anti-SBV Gc antibody (1:1000) at room temperature for 1 h, followed by goat anti-mouse IgG-HRP secondary antibody (1:1000) for 1 h. Protein bands were detected using a chemiluminescence imaging system (Azure C300-C600, Azure Biosystems, Dublin, CA, USA).

To detect bovine anti-SBV antibodies, after infection, the medium was replaced with DMEM containing 2% FBS and incubated for 36 h. The supernatant was collected, clarified by centrifugation at 4000× *g* to remove cell debris, and the virus was purified by ultracentrifugation at 80,000× *g*. The viral pellet was resuspended in 100 μL cold PBS. The viral sample was then subjected to SDS-PAGE, followed by membrane transfer. After blocking, the membrane was incubated with bovine SBV-positive serum (1:200) at room temperature for 1 h, followed by incubation with goat anti-bovine IgG-HRP secondary antibody (1:1000) for 1 h. Protein bands were detected using the chemiluminescence imaging system.

### 2.6. Indirect Immunofluorescence Assay (IFA)

Vero cells were cultured in DMEM supplemented with 10% FBS. Once the cell density reached 40–50% confluence in confocal dishes, the cells were infected with either rVSVΔG-eGFP-SBVGPC or rVSV-eGFP at an MOI of approximately 0.01. After 36 h of infection, the culture medium was removed, and the cells were fixed with 4% paraformaldehyde at room temperature for 20 min. Following fixation, the cells were washed three times with PBST, treated with 0.5% Triton X-100 for 15 min, and washed three additional times with PBST. The cells were then incubated with mouse polyclonal antibodies against SBV Gc (1:1000) at room temperature for 1 h, followed by three washes with PBST. The secondary antibody, Alexa Fluor 594-conjugated goat anti-mouse IgG (1:200), was applied and incubated for 1 h. After three additional washes with PBST, the cell nuclei were stained with DAPI. Finally, the cells were suspended in PBS buffer and analyzed for fluorescence using a confocal laser scanning microscope with Airyscan LSM800 (Zeiss, Jena, Germany).

### 2.7. Transmission Electron Microscopy

The recombinant viruses, rVSVΔG-eGFP-SBVGPC and rVSV-eGFP, were purified and prepared for transmission electron microscopy (TEM) analysis using the phosphotungstic acid negative staining. This method facilitates the visualization of viral particles by staining them with phosphotungstic acid, which creates a clear contrast between the virus particles and the surrounding background. The morphology of the virus particles was then observed using a TEM (Tokyo, Japan) to assess their size, shape, and overall structure.

### 2.8. Determination of Viral Growth Curve

Vero cells (4 × 10^6^) were seeded in T75 flasks and infected with either rVSVΔG-eGFP-SBVGPC or rVSV-eGFP at a multiplicity of infection (MOI) of 0.01. After 1 h of incubation, the cells were washed three times with PBS, then replaced with DMEM containing 2% FBS. The cells were incubated at 37 °C with 5% CO_2_, and supernatants were collected every 12 h to monitor viral propagation.

Viral titers were determined using the 50% tissue culture infectious dose (TCID_50_) assay. Briefly, the collected supernatants were serially diluted 10-fold in DMEM and inoculated into 96-well plates containing Vero E6 cells at 90% confluence, with eight replicate wells for each dilution. After 1 h of infection, the viral inoculum was removed, cells were washed three times with PBS, and replaced with DMEM containing 2% FBS. The plates were then incubated at 37 °C with 5% CO_2_ for 84 h. Fluorescence signals in the cells were observed under a microscope, and the number of wells showing fluorescence at each dilution was recorded. The viral titer was then calculated using the Reed–Muench method and expressed as TCID_50_/mL.

### 2.9. rVSVΔG-eGFP-SBVGPC Infection in Various Cells

Vero cells from monkey origin, HEK293T cells from human origin, BSR cells from Syrian hamster origin, PK-15 cells from pig origin, MDCK cells from dog origin, MDBK cells from bovine origin, MDOK cells from goat origin, and BT cells from bovine origin were infected with rVSVΔG-eGFP-SBVGPC at an MOI of 0.1. Fluorescence was observed at 36 h post-infection using the AMG EVOS FL microscope. Subsequently, the cells were fixed with 4% paraformaldehyde (PFA) (Beyotime, Zhangjiang Hi-Tech Park, Pudong New Area, Shanghai, China), resuspended in PBS, and analyzed using the Apogee A60-Universal for flow cytometry (Apogee Flow Systems, Hemel Hempstead, Hertfordshire, United Kingdom). To ensure the accuracy of flow cytometry analysis, a stepwise gating strategy was employed. First, cell populations were refined based on 488-SALS (Peak) and 488-LALS (Peak), which reflect the forward and side scatter characteristics of cells, respectively. This step excluded cellular debris and non-target cells, thereby enriching for cells with the appropriate size and granularity. Next, singlet cells were identified by plotting a scatter plot of 488-SALS (Peak) versus 488-SALS (Area), allowing for the exclusion of doublets and cell aggregates. Finally, within the gated singlet population, green fluorescence signals in the 488-Grn (Peak) channel were analyzed. Histograms of fluorescence intensity versus event count were generated to assess the proportion and distribution of GFP-positive cells. The threshold for GFP positivity was determined based on uninfected negative control samples. This gating strategy (Supplementary Figure S1A) was consistently applied across all experimental groups to ensure data comparability and consistency.

### 2.10. Virus Neutralization Test (VNT)

To evaluate the neutralization capacity of the recombinant virus rVSVΔG-eGFP-SBVGPC, we used three types of serum samples: commercially available freeze-dried SBV-positive bovine serum (MRI-SBV, Innovative Diagnostics, Grabels, France), as well as sera from immunized mice and calves. The immune sera were collected from five 7-week-old SPF female BALB/c mice and five 6-month-old calves, all immunized twice with the SBV protective antigen Gc expressed using the Expi293F system.

We first cloned the gene sequence encoding the extracellular region of the SBV Gc protein into the pCAGGS eukaryotic expression vector and transfected it into Expi293F suspension cells for protein expression. The culture supernatant was collected, and the Gc protein was purified via affinity chromatography. The purified protein was then mixed with ISA 15A VG adjuvant to prepare the immunogen. The immunogen was administered to the mice at a dose of 20 µg per mouse, followed by a booster immunization at the same dose three weeks later. Serum was collected two weeks after the booster and used as mouse immune serum in the recombinant virus neutralization assays. The immunization protocol for the calves was similar, with each calf receiving 150 µg of the immunogen, followed by a booster three weeks later. Serum was collected two weeks after the booster for use in the neutralization assays. All serum samples were heat-inactivated at 56 °C for 30 min prior to testing, then subjected to two-fold serial dilutions up to a maximum dilution of 1:2^14^. Eight replicates were prepared for each dilution. The diluted sera were mixed with an equal volume of virus and incubated at 37 °C for 1 h. Then, 50 μL of the virus-serum mixture (final dose: 100 TCID_50_ of rVSVΔG-eGFP-SBVGPC per well) was added to 96-well plates pre-seeded with Vero cells and incubated at 37 °C with 5% CO_2_ for 1 h. After incubation, DMEM containing 10% fetal bovine serum (FBS) was added to each well, and the cells were further incubated under the same conditions for 48 h. At the end of the incubation, cells were harvested by trypsinization, washed twice with PBS, and resuspended in PBS. Replicates from the same dilution were pooled into one tube for analysis by a multiparameter flow cytometer. The percentage of green fluorescent-positive cells was measured, and the relative virus infection rate was calculated accordingly. The neutralizing antibody titer (NT_50_) was determined using the Reed–Muench method. In each experiment, fetal bovine serum (FBS) was used as a negative control, with its infection rate set as the baseline (100%). The relative infection rates of all positive samples were calculated against this reference to ensure the accuracy and comparability of the experimental results.

Additionally, to assess the specificity of rVSVΔG-eGFP-SBVGPC in serological detection, neutralization assays were performed using bovine sera positive for BRV, BHV-1, BCoV, BLV, and BVDV, which were stored in our laboratory. Each serum sample was serially diluted from 1:2^1^ to 1:2^3^. Eight replicates were set up for each dilution. The diluted sera were mixed with an equal volume of virus (100 TCID_50_ per well) and incubated at 37 °C for 1 h. Then, 50 μL of the mixture was added to 96-well plates pre-seeded with Vero cells. After incubation at 37 °C with 5% CO_2_ for 1 h, DMEM medium containing 10% FBS was added to each well, and the plates were further incubated for 48 h before observation.

### 2.11. Statistical Analysis

The differences in particle sizes between the recombinant virus and rVSV-eGFP were analyzed using an unpaired *t*-test. To evaluate the differences in viral titers between the two viruses at different time points, the normality of the data distribution was first assessed using the Shapiro–Wilk test. An independent samples *t*-test was then performed to compare the peak titers of the two viruses, and analysis of variance (ANOVA) was applied to analyze the replication kinetics. All statistical analyses were performed using GraphPad Prism 9, with a *p*-value of < 0.05 considered statistically significant.

## 3. Results

### 3.1. Rescue of Replication-Competent rVSV Expressing SBV Envelope Glycoproteins

**Figure 1:**
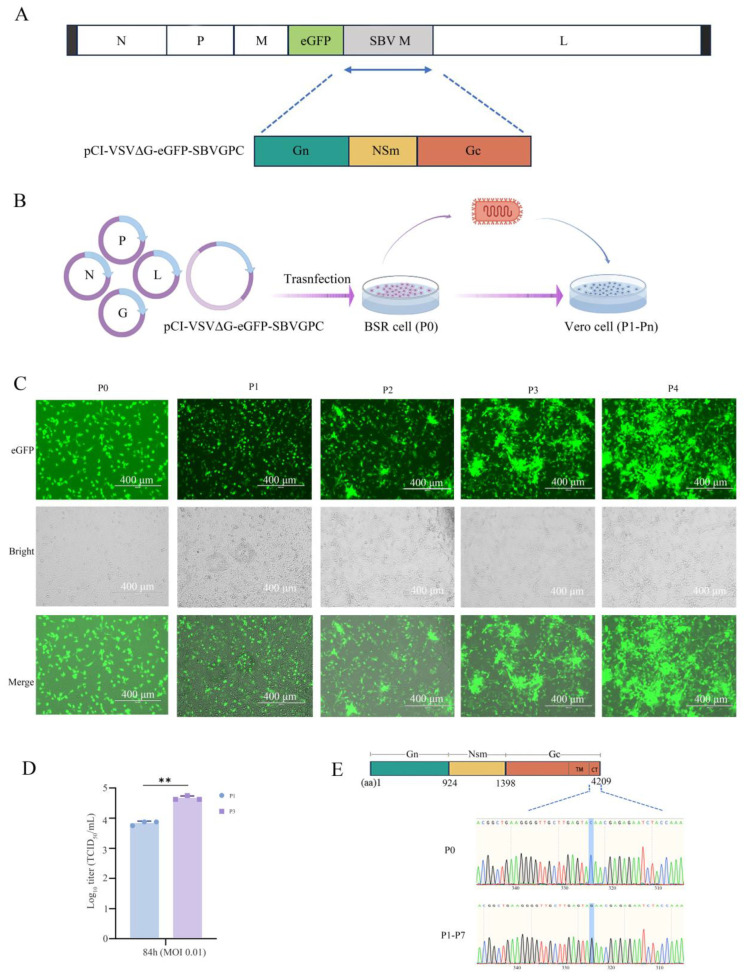
Construction and rescue of rVSVΔG-eGFP-SBVGPC. (**A**) Genomic organization of rVSVΔG-eGFP-SBVGPC. Viral genome is shown in a 3′-to-5′ orientation. Genes N (nucleocapsid), P (phosphoprotein), M (matrix), and L (large polymerase) are conserved from VSV. The VSV G gene is replaced by the SBV M segment, which encodes Gn, NSm, and Gc proteins. Enhanced green fluorescent protein (eGFP) is inserted upstream of the SBV M gene as a marker of infection. (**B**) Schematic representation of the generation process. The recombinant plasmid pVSVΔG-eGFP-SBVGPC, along with helper plasmids encoding VSV N, P, L, and G proteins, was transfected into BSR cells. The supernatant containing the P0 virus was collected and used to infect Vero cells for subsequent passages. (**C**) Fluorescence detection and cytopathic effect (CPE) during viral passages. Green fluorescence images show the progressive increase in infection efficiency and eGFP expression from P0 to P4, with corresponding bright-field images revealing cytopathic effects. Scale bars represent 400 μm. (**D**) Replication efficiency and passage stability of rVSVΔG-eGFP-SBVGPC in BSR cells. Viral titers of first passage (P1) and third passage (P3) viruses were measured at 84 h post-infection at an MOI of 0.01. Data are presented as mean ± SD from three independent experiments. **, *p <* 0.01. (**E**) Identification of a nonsense mutation in the Gc gene of rVSVΔG-eGFP-SBVGPC during serial passage. Sanger sequencing revealed a nonsense mutation (TAC→TAG) at codon 925 of the Gc protein, resulting in premature termination of translation. This mutation was consistently maintained in subsequent viral passages.

To construct a replication-competent recombinant vesicular stomatitis virus (rVSV) expressing the envelope glycoproteins of Schmallenberg virus (SBV), the G gene in the VSV genome was replaced with the M gene segment of SBV. Additionally, an enhanced green fluorescent protein (eGFP) gene was inserted upstream of the M gene segment to facilitate visual monitoring of viral replication. The modified full-length viral genome and its regulatory elements were cloned into an expression vector to generate the recombinant plasmid pCI-VSVΔG-eGFP-SBVGPC (Figure 1A).

The plasmid pCI-VSVΔG-eGFP-SBVGPC was co-transfected with the helper plasmids pCI-VSV-N, -P, -L, and -G into BSR cells. When more than 90% of the cells displayed green fluorescence, the supernatant (P0) was collected and passed into Vero cells for amplification. During subsequent passages, enhanced fluorescence and more pronounced cytopathic effects (CPE) were observed, indicating the successful rescue and stable propagation of the recombinant virus (Figure 1C).

To evaluate the replication efficiency and passage stability, BSR cells were infected with first (P1) and third (P3) passage viruses at a multiplicity of infection (MOI) of 0.01, and viral titers were measured at 84 h post-infection. The results showed that the viral titer of the P3 virus was significantly higher than that of the P1 virus (*p* < 0.01), indicating that the recombinant virus maintained good genetic stability and exhibited adaptive replication enhancement during serial passages (Figure 1D). Furthermore, sequencing analysis of the serially passaged viruses revealed a nonsense mutation (TAC→TAG) at codon 925 of the Gc protein, resulting in premature translational termination, which persisted in subsequent passages (Figure 1E). These findings suggest that the VSV vector system may undergo adaptive selection when expressing heterologous viral glycoproteins, consistent with similar observations reported previously [29,30].

### 3.2. Characterization of rVSVΔG-eGFP-SBVGPC

**Figure 2:**
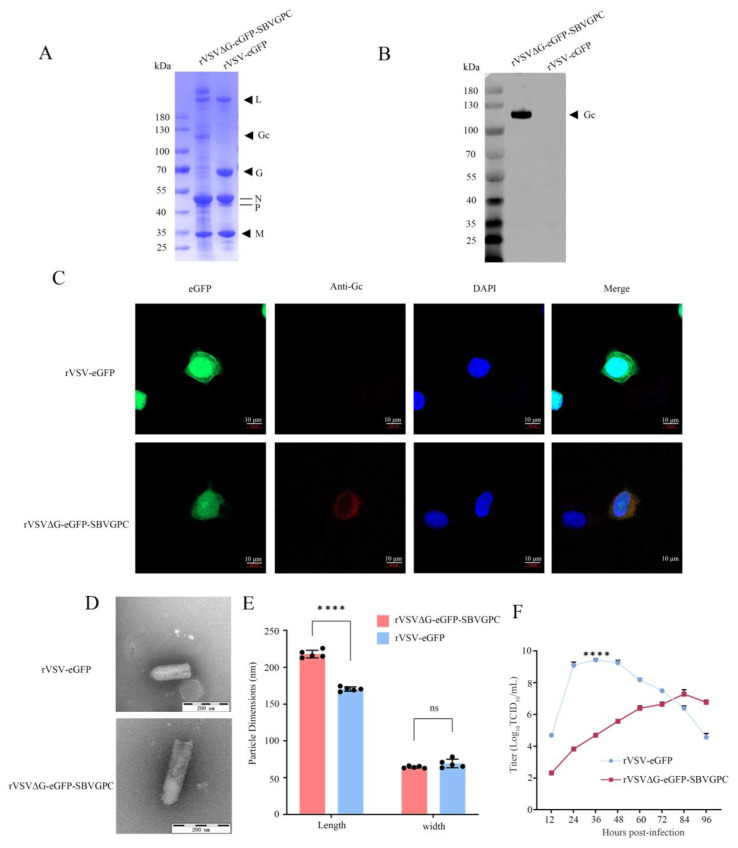
Characterization of rVSVΔG-eGFP-SBVGPC. (**A**) SDS-PAGE analysis of purified rVSVΔG-eGFP-SBVGPC (Lane 1) and rVSV-eGFP (Lane 2) virions stained with Coomassie blue. Positions of viral proteins, including Gc, are indicated on the right. (**B**) Western blot analysis of purified virions. rVSVΔG-eGFP-SBVGPC virions reacted specifically with mouse anti-Gc protein serum (Lane 1), while no reactivity was observed for rVSV-eGFP virions (Lane 2). (**C**) Immunofluorescence analysis showing expression of SBV Gc protein in Vero cells infected with rVSVΔG-eGFP-SBVGPC. Cells were stained with mouse anti-Gc protein serum followed by Alexa-Fluor 594-labeled secondary antibody. Red fluorescence indicates Gc expression. No red fluorescence was observed in cells infected with rVSV-eGFP. Scale bar = 10 μm. (**D**) Transmission electron micrographs of purified virions stained with 2% phosphotungstic acid (PTA). rVSVΔG-eGFP-SBVGPC virions displayed surface spikes, consistent with incorporation of SBV glycoproteins. (**E**) Measurements of viral particle dimensions based on electron micrographs. Length of rVSVΔG-eGFP-SBVGPC particles was significantly greater than that of rVSV-eGFP particles, while no significant difference in particle width was observed. Data are presented as mean ± standard deviation (*n* = 5). ****, *p <* 0.0001. (**F**) Growth kinetics of rVSVΔG-eGFP-SBVGPC and rVSV-eGFP in Vero cells. Viral titers (TCID_50_/mL) were measured at various time points post-infection. ****, *p <* 0.0001.

To further characterize the rescued rVSVΔG-eGFP-SBVGPC, the recombinant virus was purified and concentrated using density gradient centrifugation and analyzed by SDS-PAGE, with the parental rVSV-eGFP virus used as a control. SDS-PAGE analysis revealed a band corresponding to the expected molecular weight of the Gc protein in the recombinant virus (Figure 2A; see Supplementary Materials). Additionally, Western blotting demonstrated specific reactivity of the recombinant virus with mouse anti-Gc serum (Figure 2B; see Supplementary Materials). Confocal laser microscopy further confirmed the expression of Gc, showing that cells infected with the recombinant virus rVSVΔG-eGFP-SBVGPC exhibited specific binding to mouse anti-Gc serum, resulting in red fluorescence. In contrast, Vero cells infected with rVSV-eGFP did not express Gc, and no red fluorescence was observed (Figure 2C). These findings confirmed that the SBV envelope glycoprotein Gc was properly expressed in the recombinant virus rVSVΔG-eGFP-SBVGPC. It is noteworthy that due to the unavailability of Gn-specific antibodies, the expression of the Gn protein could not be directly characterized in this study. Nevertheless, full-length sequencing of the recombinant virus genome verified the successful integration of the Gn gene, and the autonomous replication and infection phenotype of the virus indirectly support its functional expression.

To investigate the morphological characteristics of the recombinant virus, electron microscopy samples of the purified recombinant virus rVSVΔG-eGFP-SBVGPC were prepared using tungsten phosphotungstate negative staining. Transmission electron microscopy revealed that the virus particles displayed spikes on their surface, consistent with the incorporation of SBV glycoproteins (Figure 2D). The insertion of the SBV M segment open reading frame (ORF) introduced approximately 2500 nucleotides into the VSV genome. As a result, rVSVΔG-eGFP-SBVGPC particles were significantly longer than those of wild-type VSV (*p* < 0.0001), although no significant difference in particle width was observed (Figure 2E).

To compare the growth kinetics of the recombinant virus rVSVΔG-eGFP-SBVGPC with the parental rVSV-eGFP, both viruses were inoculated into Vero cells, and viral titers (TCID_50_) were measured to generate growth curves. The results showed that the peak titer of the recombinant virus at 84 h post-infection (10^7^ TCID_50_/mL) was significantly lower than that of rVSV-eGFP at 36 h post-infection (10^9^ TCID_50_/mL) (*p* < 0.0001) (Figure 2F). These results indicate that the substitution of the SBV glycoproteins has a significant effect on viral replication dynamics.

### 3.3. Host Cell Tropism of rVSVΔG-eGFP-SBVGPC

**Figure 3:**
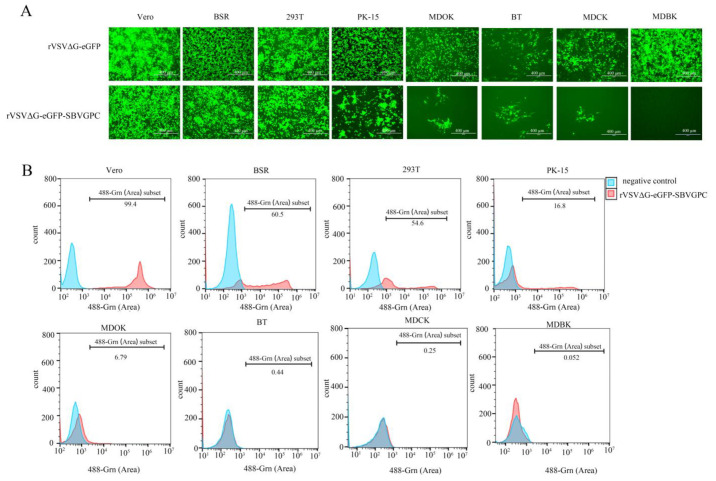
Infection of rVSVΔG-eGFP-SBVGPC in different host cells. (**A**) Infection of different cell lines with rVSVΔG-SBVGPC (MOI 0.1) and rVSV-eGFP (MOI 0.01). Fluorescence was observed 36 h post-infection using an inverted fluorescence microscope. Scale bar represents 400 µm. (**B**) Flow cytometric analysis of rVSVΔG-eGFP-SBVGPC infection in different cell lines. Flow cytometry data were analyzed using FlowJo software (Version 10.8.1, Becton Dickinson, USA), and infection levels were quantitatively assessed.

The recombinant virus rVSVΔG-eGFP-SBVGPC replaced the G protein of rVSV-eGFP, which is responsible for mediating viral entry, with the envelope glycoproteins of Schmallenberg virus (SBV). To evaluate the cell tropism of this recombinant virus, it was inoculated into eight cell lines derived from different species, including bovine-derived MDBK and BT cells, ovine-derived MDOK cells, canine-derived MDCK cells, porcine-derived PK-15 cells, murine-derived BSR cells, human-derived HEK293T cells, and monkey-derived Vero cells (Figure 3A). The results showed that rVSVΔG-eGFP-SBVGPC altered the tropism profile of the parental virus across these cell lines. We further analyzed the infection efficiency using flow cytometry. The results showed that Vero cells were the most susceptible to rVSVΔG-eGFP-SBVGPC, with an infection rate of 99.4%, followed by BSR (60.5%), HEK293T (54.6%), PK-15 (16.8%), and BT cells (6.79%). In contrast, MDCK and MDOK cells exhibited infection rates below 1%, and no infection was detected in MDBK cells (Figure 3B). These findings are consistent with the known broad host range of SBV [5]. Taken together, the results indicate that rVSVΔG-eGFP-SBVGPC acquires the host cell tropism of SBV by utilizing SBV envelope glycoproteins to mediate membrane fusion and viral entry. Therefore, rVSVΔG-eGFP-SBVGPC can serve as an effective model virus for studying the mechanisms of SBV entry into different host cells.

### 3.4. Detection of SBV Neutralizing Antibodies Using rVSVΔG-eGFP-SBVGPC

**Figure 4:**
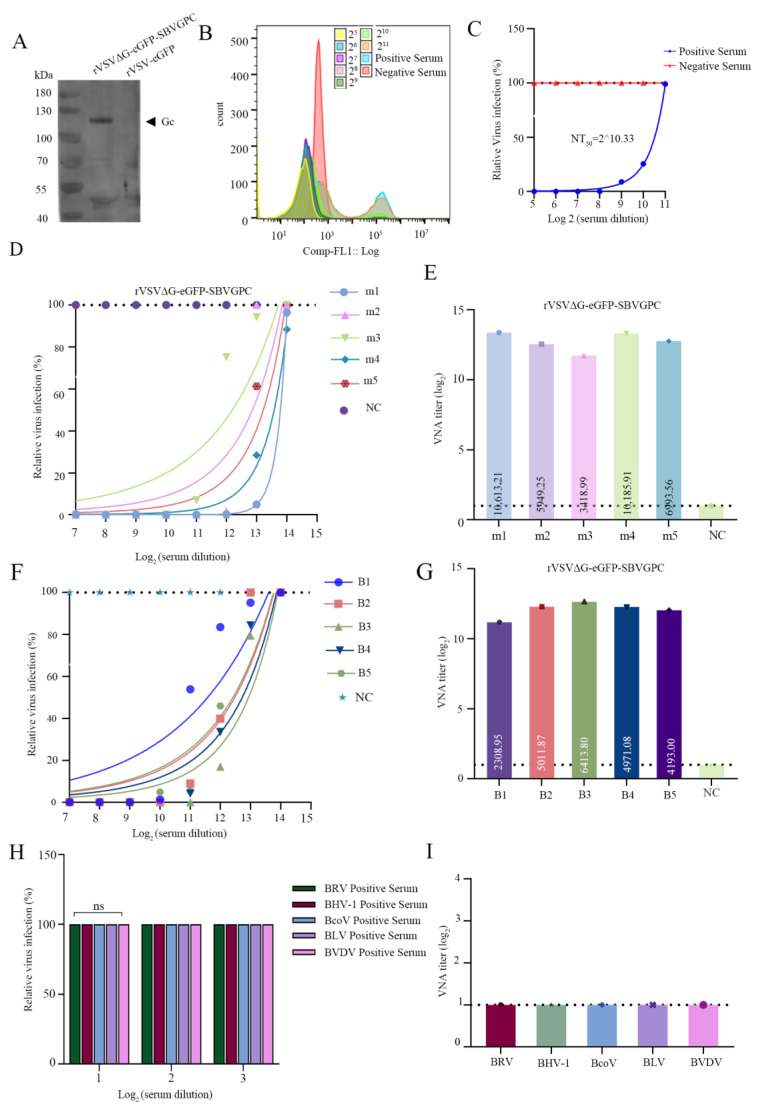
VNT using rVSVΔG-eGFP-SBVGPC to detect SBV neutralizing antibodies. (**A**) Western blot analysis of purified rVSVΔG-eGFP-SBVGPC (Lane 1) and purified rVSV-eGFP (Lane 2) using SBV-positive serum. (**B**,**C**) Serum neutralizing antibody titers against rVSVΔG-eGFP-SBVGPC were determined using a virus neutralization test (VNT). SBV-positive serum was serially diluted and mixed with rVSVΔG-eGFP-SBVGPC, followed by incubation to ensure complete neutralization. The mixture was then added to Vero cells in a 96-well plate for further incubation. GFP expression in the cells was detected using a multiparameter flow cytometer (Apogee) to assess viral infection (**B**), and the relative virus infection rate was calculated using negative serum as the baseline (**C**). The neutralizing antibody titer (NT_50_) was defined as the highest serum dilution that completely inhibited GFP expression in 50% of the wells, and was calculated using the Reed–Muench method. (**D**) Flow cytometry was used to analyze the inhibition of rVSVΔG-eGFP-SBVGPC infection by polyclonal antisera against SBV Gc obtained from immunized mice (m1–m5), with fetal bovine serum (FBS) as the negative control. (**E**) Neutralizing antibody titers of mouse sera (m1–m5) shown in panel D were quantified based on flow cytometry. (**F**) Flow cytometry was used to analyze the inhibition of rVSVΔG-eGFP-SBVGPC infection by polyclonal antisera against SBV Gc obtained from immunized cattle (B1–B5), with FBS as negative control. (**G**) Neutralizing antibody titers of cattle sera (B1–B5) shown in panel F were quantified using flow cytometry. (**H**) Flow cytometry was used to analyze the inhibitory effects of bovine virus-specific sera (BRV, BHV-1, BCoV, BLV, and BVDV) on rVSVΔG-eGFP-SBVGPC infection, with FBS as the negative control. (**I**) Neutralizing antibody titers of various heterologous bovine virus-positive sera shown in panel H were quantified based on flow cytometry.

To evaluate the potential of the recombinant virus rVSVΔG-eGFP-SBVGPC to replace the traditional SBV strain in serological detection, we first performed Western blot analysis using freeze-dried serum positive for SBV from naturally infected cattle. The results showed that specific antibodies in the SBV-positive serum recognized the relevant proteins expressed by the recombinant virus, with a clear band appearing at approximately 110 kDa, corresponding to the SBV Gc glycoprotein (Figure 4A; see Supplementary Materials). This indicates that rVSVΔG-eGFP-SBVGPC successfully expresses the key antigenic components and can be specifically recognized by antibodies generated after SBV infection, demonstrating its potential for serological detection.

Next, we assessed the neutralizing antibody detection capability of the recombinant virus using serial dilutions (from 1:2^5^ to 1:2^11^) of SBV-positive bovine serum. The results showed that at low dilutions (1:2^5^ to 1:2^7^), the serum completely neutralized the recombinant virus infection, demonstrating strong neutralizing activity. As the dilution increased (≥1:2^8^), the neutralizing effect gradually weakened, and the viral infection rate rose stepwise. At a 1:2^11^ dilution, the infection rate returned to a level comparable to the negative control group (Figure 4B). The neutralizing antibody titer calculated using the Reed–Muench method was 2^10.33^ (Figure 4C). These results further confirm the effectiveness of this system for detecting SBV-neutralizing antibodies.

To expand the applicability of the detection platform, we further conducted neutralization assays using sera from mice and cattle immunized with the SBV Gc protein. Flow cytometry results showed that with increasing serum dilution, the inhibitory effect of the immune serum on recombinant virus infection gradually decreased, as evidenced by a stepwise increase in the proportion of eGFP-positive cells. Quantification of the positive cell proportion allowed accurate determination of the neutralizing antibody titers in the immune sera (Figure 4D–G). In contrast, the negative control fetal bovine serum (FBS) showed no neutralizing activity, with the infection rate of the recombinant virus remaining consistently at 100%.

To further verify the specificity of the system, neutralization assays were performed using positive sera against various heterologous bovine viruses (including BRV, BHV-1, BCoV, BLV, and BVDV) at dilutions from 1:2^1^ to 1:2^3^. The results showed that these sera did not inhibit recombinant virus infection, and infection rates did not differ significantly from those of the FBS negative control group (Figure 4H–I), fully demonstrating the high specificity of this detection system in identifying SBV neutralizing antibodies.

In summary, the detection system constructed with recombinant virus rVSVΔG-eGFP-SBVGPC can sensitively, specifically, and quantitatively detect SBV neutralizing antibodies in clinical or immunized animal sera. Therefore, this platform holds significant advantages for vaccine immunogenicity evaluation and epidemic surveillance, showing broad prospects for replacing traditional SBV strains in serological testing.

## 4. Discussion

Since the emergence of SBV in 2011, it has rapidly spread across Europe, significantly impacting the economic viability of livestock farming [31,32]. With the increasing development of global animal trade, the pressure of viral transmission into SBV non-endemic regions has grown, creating an urgent need for scientifically reliable monitoring methods. To overcome the limitation of the absence of an SBV strain for diagnostic and vaccine research, this study employed a reverse genetics system to construct a recombinant VSV virus expressing the SBV envelope glycoprotein. This approach provides a reliable tool for SBV seroepidemiological surveillance, as well as for the development and evaluation of novel vaccines.

In constructing the recombinant virus genome, we explored several strategies, including the construction of plasmids encoding SBV Gn and SBV Gc separately, Gn and Gc fusion plasmids, and a plasmid containing the complete M gene segment encoding SBV GPC (). Ultimately, we found that only replacing the VSV G protein with the complete GCP successfully rescued the recombinant virus. This indicates that the full GCP is essential for the assembly and infection of SBV. After rescuing the rVSVΔG-eGFP-SBVGPC, we observed an increase in the virus’s infectivity in successive passages. Specifically, the CPE became more pronounced, and the green fluorescence signal grew stronger, likely due to adaptive mutations in VSV that enhance viral infectivity. The improved cellular adaptation ensures that the virus will maintain sensitivity as a serological detection tool. Notably, although rVSVΔG-eGFP-SBVGPC induced evident cytopathic effects (CPE) in BSR cells, including cell rounding, detachment, and apoptosis, no syncytium formation was observed. This phenotype is consistent with that of wild-type SBV, whose Gc protein mediates pH-dependent (pH 5.0–6.0) endosomal membrane fusion rather than direct fusion at the plasma membrane, further supporting the biological relevance of this model for SBV pathogenesis studies [33]. In addition, sequencing analysis of serially passaged viruses revealed a nonsense mutation (TAC→TAG) at codon 925 of the Gc protein, resulting in premature translational termination, which persisted in subsequent passages. This finding suggests that adaptive selection may occur when the VSV vector system expresses heterologous viral glycoproteins. Similar adaptive mutations have been reported in previous studies [29,30], indicating that the expression of foreign envelope proteins in the VSV system may follow common evolutionary patterns. These observations warrant further investigation into the underlying molecular mechanisms and their impact on viral phenotypes.

Following the successful rescue of the rVSVΔG-eGFP-SBVGPC virus, the virus was characterized, with a focus on the expression of the envelope glycoprotein Gc, its morphological features, and growth kinetics. The SBV GPC comprises three proteins: Gn, Nsm, and Gc [34,35,36]. The Gc glycoprotein is the key mediator of virus-cell fusion, promoting viral infection and serving as the primary target for the induction of neutralizing antibodies [37]. Using IFA and Western blot, we confirmed that Gc was successfully expressed in the recombinant virus. However, due to the lack of specific sera for Gn and Nsm, the expression of these proteins has not yet been detected. Nevertheless, based on the virus’s infectivity and replication characteristics, it can be inferred that Gn and Nsm were also successfully expressed and functional. Additionally, due to the replacement of the VSV envelope glycoprotein, the virus underwent significant morphological changes. Electron microscopy images revealed spike-like structures characteristic of the SBV envelope glycoprotein. Because of the notable difference in molecular weight between these proteins and the VSV G protein, the recombinant virus was significantly longer than the parent virus. Although the recombinant virus exhibited slightly weaker growth and replication capabilities compared to the parent virus, its viral titer of 10^7^ TCID_50_/mL is sufficient for use as a model virus tool, including for SBV serological monitoring. These findings confirm that the recombinant virus was successfully constructed, rescued, and characterized.

The envelope glycoproteins of SBV play a crucial role in mediating viral infection of host cells. Therefore, we assessed the infectivity of the recombinant virus in cells from different species. Our results demonstrate that the cell tropism of the recombinant virus is consistent with the previously reported tropism of SBV, showing high sensitivity to Vero cells, BSR cells, and porcine kidney cells [38]. Although evidence suggests that SBV typically does not infect human cells, our observation of infection in 293T cells indicates that SBV may have the potential to adapt to human cells under certain conditions. This finding underscores the possible cross-species transmission capability of SBV, suggesting a potential risk to human health, particularly in environments where there is close contact with infected animals. For bovine-derived cells, the recombinant virus infects BT cells but not MDBK cells, suggesting that cell type plays a crucial role in the specificity and selectivity of SBV infection. Therefore, the recombinant VSV virus, by expressing the SBV envelope glycoprotein, acquires the infection characteristics of SBV for cells, demonstrating the potential to substitute for the SBV strain in studies such as neutralizing antibody detection, virus adsorption, and invasion.

In the application of rVSVΔG-eGFP-SBVGPC for SBV serological testing, the recombinant virus demonstrated good reactivity with SBV-positive serum from naturally infected cows. It successfully determined the neutralizing antibody titer of the serum. It also exhibited strong sensitivity and specificity in detecting neutralizing activity in immune sera targeting the SBV Gc protein. These results validated the applicability of the VNT system constructed with the recombinant virus for detecting SBV neutralizing antibodies. This method utilizes eGFP fluorescence labeling combined with flow cytometry to analyze viral infection levels quantitatively. Compared with traditional CPE visual scoring, it significantly enhances detection sensitivity and objectivity, offers high throughput capability, effectively reduces human error, and is suitable for rapid screening of large sample volumes and clinical applications. Furthermore, the recombinant virus-based VNT system mitigates the high biosafety risks associated with wild-type SBV, thereby significantly improving experimental safety and operability. This detection platform holds promising prospects for widespread application in SBV non-endemic regions and can serve as an effective tool for serological surveillance and evaluation of vaccine immunogenicity. Future work will focus on further optimizing the system to enhance its practicality and clinical translation value.

This study successfully constructed and functionally validated the recombinant VSV-based chimeric virus rVSVΔG-eGFP-SBVGPC, which expresses the envelope glycoprotein of Schmallenberg virus (SBV). By expressing the SBV Gc protein and acquiring SBV-specific cell tropism, this recombinant virus can be used to detect neutralizing antibody titers, demonstrating promising potential for clinical diagnostic applications and overcoming critical limitations in serological surveillance and vaccine development in non-endemic regions due to the lack of virus strains. Moreover, this recombinant virus serves as an important model for studying SBV infection mechanisms, cell tropism, cross-species transmission potential, and immune evasion strategies. Future optimizations and expanded applications will further enhance its value in SBV outbreak control, vaccine development, and antiviral research.

## Data Availability

Data are available on request from corresponding authors.

## Supplementary Materials

The following supporting information can be downloaded at: https://www.mdpi.com/article/10.3390/vetsci12090809/s1, File S1: Original WB blots used for analysis in Figure 2A, Figure 2B, and Figure 4A; File S2: Experimental supplementary Figure S1.
