# Supplementary material for: Construction and Characterization of a Vesicular Stomatitis Virus Chimera Expressing Schmallenberg Virus Glycoproteins
Source: Vet Sci. 2025 Aug 25;12(9):809. doi: 10.3390/vetsci12090809 (PMC12474314; doi:10.3390/vetsci12090809)
Supplement: Supplementary file 1 [file vetsci-12-00809-s001.zip › File S1 Original WB images.pdf]

Original WB images for Figure 2A

M 1 2

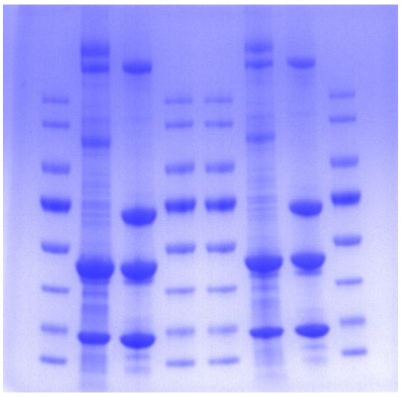

SDS-PAGE analysis of purified rVSVΔG-eGFP-SBVGPC (Lane 1) and rVSV-eGFP (Lane 2) virions stained with Coomassie blue. The positions of viral proteins, including Gc, are indicated on the right.

Original WB images for Figure 2B

M 1 2

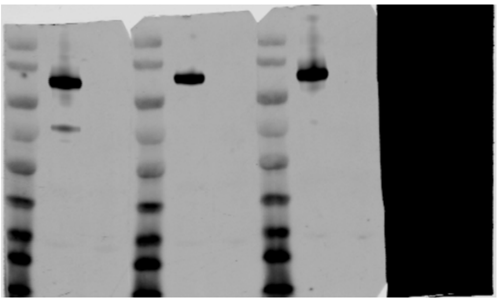

Western blot analysis of purified virions. The rVSVΔG-eGFP-SBVGPC virions reacted specifically with mouse anti-Gc protein serum (Lane 1), while no reactivity was observed for rVSV-eGFP virions (Lane 2).

Original WB images for Figure 4A

M 1 2

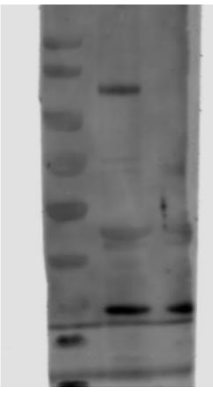

Western blot analysis of purified rVSVΔG-eGFP-SBVGPC (Lane 1) and purified rVSV-eGFP

(Lane 2) using SBV-positive serum
