# Supplementary material for: Construction and Characterization of a Vesicular Stomatitis Virus Chimera Expressing Schmallenberg Virus Glycoproteins
Source: Vet Sci. 2025 Aug 25;12(9):809. doi: 10.3390/vetsci12090809 (PMC12474314; doi:10.3390/vetsci12090809)
Supplement: Supplementary file 1 [file vetsci-12-00809-s001.zip › File S2 Experimental supplementary Figure S1.pdf]

Supplementary Materials

Construction and Characterization of a Vesicular Stomatitis Virus Chimera Expressing Schmallenberg Virus Glycoproteins

Huijuan Guo †, Zhigang Jiang†, Jing Wang, Fang Wang, Qi Jia, Zhigao Bu *, Xin Yin * and Zhiyuan Wen *

State Key Laboratory for Animal Disease Control and Prevention, Harbin Veterinary Research Institute,
Chinese Academy of Agricultural Sciences, Harbin 150069, China;
18738046032@163.com (H.G.);
jiangzhigang@caas.cn (Z.J.); wangjing_19940223@163.com (J.W.);
wangfang06@caas.cn (F.W.);
jiaqivet@163.com (Q.J.)

* Correspondence: buzhigao@caas.cn (Z.B.); yinxin@caas.cn (X.Y.); wenzhiyuan@caas.cn (Z.W.)

† These authors contributed equally to this work.

Figure S1. Flow cytometry gating strategy and time course of cytopathic effects (CPE) induced by rVSVΔG-eGFP-SBVGPC.

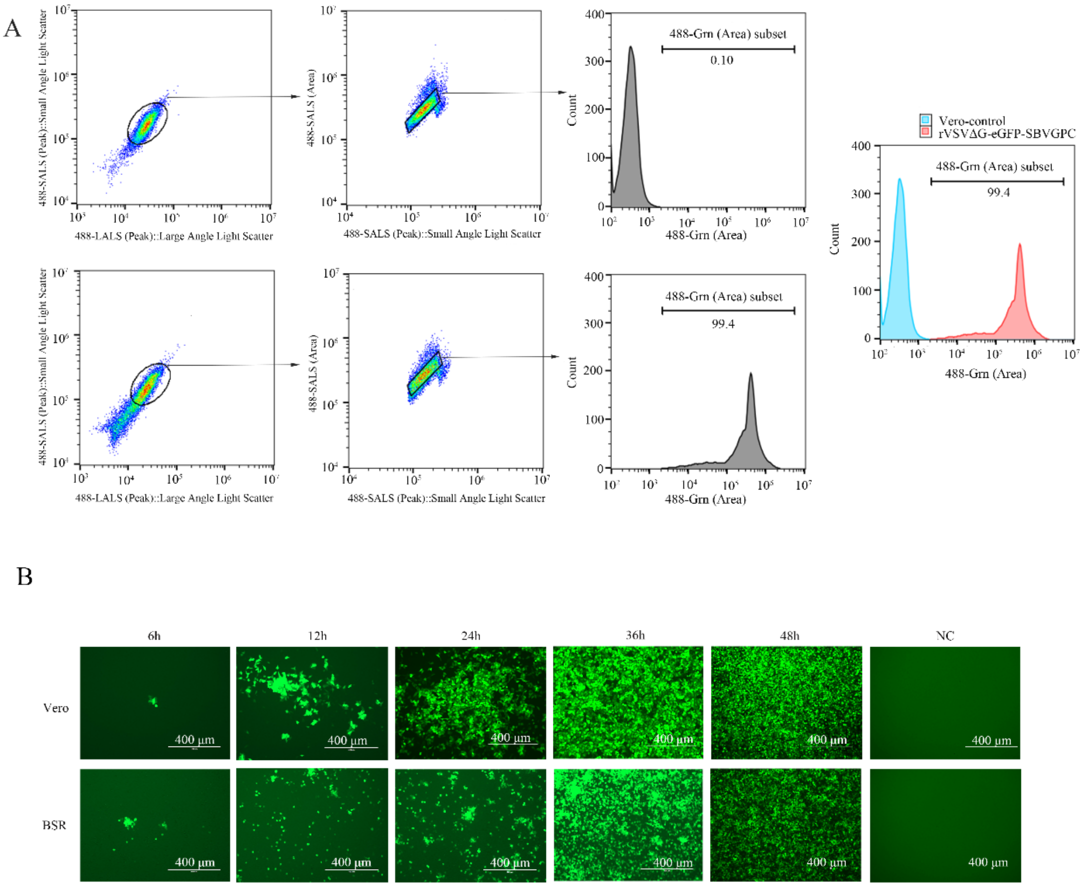

(A) Gating strategy for flow cytometric analysis of Vero cells infected with rVSVΔG-eGFP-SBVGPC. Debris and non-target events were first excluded based on 488-SALS (Peak) versus 488-LALS (Peak) (left panel), followed by the selection of singlets via 488-SALS (Peak) versus 488-SALS (Area) (middle left panel). GFP-positive cells were then identified by analyzing green fluorescence in the 488-Grn (Peak) channel (middle right panel). The upper row shows uninfected Vero cells (negative control), while the lower row shows infected cells. The rightmost panel presents an overlay of the fluorescence histograms from both groups to illustrate the shift in GFP signal. (B) Time course of cytopathic effects (CPE) induced by rVSVΔG-eGFP-SBVGPC in Vero and BSR cells. Cells were infected at an MOI of 0.1, and GFP fluorescence and CPE were monitored at 6, 12, 24, 36, and 48 hours post-infection. Scale bar = 400 μm.
